# Factors related to HPV vaccine uptake and 3-dose completion among women in a low vaccination region of the USA: an observational study

**DOI:** 10.1186/s12905-016-0323-5

**Published:** 2016-07-22

**Authors:** Andrew R. Wilson, Mia Hashibe, Julia Bodson, Lisa H. Gren, Brooke A. Taylor, Jessica Greenwood, Brian R. Jackson, Rosemary She, Marlene J. Egger, Deanna Kepka

**Affiliations:** University of Utah, College of Nursing, 10 South 2000 East, Room 2200, Salt Lake City, Utah 84112 USA; Huntsman Cancer Institute, Cancer Control and Population Sciences, 2000 Circle of Hope, Salt Lake City, Utah 84112 USA; University of Utah, Department of Family and Preventive Medicine, University of Utah School of Medicine, 375 Chipeta Way Ste, A Salt Lake City, Utah 84108 USA; ARUP Laboratories, 500 Chipeta Way, Salt Lake City, Utah 84108 USA; Department of Pathology, University of Utah, 15 North Medical Drive East, Salt Lake City, Utah 84112 USA; University of Southern California, Keck School of Medicine, 1975 Zonal Ave, Los Angeles, California 90033 USA

**Keywords:** HPV, Human Papillomavirus, Vaccination, Immunization

## Abstract

**Background:**

To assess the demographic and attitudinal factors associated with HPV vaccine initiation and completion among 18–26 year old women in Utah.

**Method:**

Between January 2013 and December 2013, we surveyed 325 women from the University of Utah Community Clinics about their HPV vaccine related beliefs and behaviors. Odds ratios (ORs) were estimated from logistic regression models to identify variables related to HPV vaccine initiation and series completion.

**Results:**

Of the 325 participants, 204 (62.8 %) had initiated the vaccine and 159 (48.9 %) had completed the 3-dose series. The variables associated with HPV vaccine initiation were lower age (OR = 1.18 per year); being unmarried (OR = 3.62); not practicing organized religion (OR = 2.40); knowing how HPV spreads (OR = 6.29); knowing the connection between HPV and cervical cancer (OR = 3.90); a belief in the importance of preventive vaccination (OR = 2.45 per scale unit); strength of doctor recommendation (OR = 1.86 per scale unit); and whether a doctor’s recommendation was influential (OR = 1.70 per scale unit). These variables were also significantly associated with HPV vaccine completion.

**Conclusion:**

The implications of these findings may help inform policies and interventions focused on increasing HPV vaccination rates among young women. For example, without this information, programs might focus on HPV awareness; however, the results of this study illustrate that awareness is already high (near saturation) in target populations and other factors, such as strong and consistent physician recommendations, are more pivotal in increasing likelihood of vaccination. Additionally, our findings indicate the need for discussions of risk assessment be tailored to the young adult population.

**Electronic supplementary material:**

The online version of this article (doi:10.1186/s12905-016-0323-5) contains supplementary material, which is available to authorized users.

## Background

The American Cancer Society estimates that in 2014, there will be over 12,000 new cases of invasive cervical cancer diagnosed in the United States and over 4,000 women will die from this disease [1]. Human papillomavirus (HPV), the most common sexually transmitted infection [2, 3], has been shown to be necessary to cause cervical cancer [4–6]. Recognition of this link led to the development of vaccines protecting against infection with certain high-risk types of HPV. There are currently two HPV vaccines available: Merck’s Gardasil® vaccine [7], proved highly effective in preventing the highest prevalence HPV types 16 and 18 (which cause up to 70 % of all cervical cancers), as well as HPV types 6 and 11 (which cause about 90 % of genital warts) [4, 8–10]; and GlaxoSmithKline’s Cervarix® vaccine [11], targeting the two most common oncogenic HPV types (16 and 18) [4, 10].

In 2006, the Centers for Disease Control (CDC) Advisory Committee on Immunization Practices (ACIP) recommended a 3-dose HPV vaccination series as a routine vaccine for girls age 11–12 years old [12]. Vaccine administration is optimal at this age because adolescents have the best immunoresponse to the vaccine and likely have not yet been exposed to the virus [12]. However, the ACIP also recommended the HPV vaccination series as a catch-up vaccine for young women age 13–26 years old [12]. Additionally, these recommendations have been extended to include males. It is hypothesized that with good vaccination coverage, the prevalence of HPV and HPV-associated cancers will decline [5, 12–14].

Despite this opportunity for cervical cancer prevention, HPV vaccination rates are low in the United States. In 2013, just 57.3 % of adolescent girls age 13–17 years old had received one dose of the vaccine, and only 37.6 % had completed the three-dose series [15]. Coverage is especially poor in Utah, with just 44.3 % of Utah adolescent girls initiating the vaccine, and 20.5 % completing the 3-dose series [15]. Most recent data indicates that uptake is the worst among young women: only 34.5 % of women age 19–26 years old report receiving at least one dose of the HPV vaccine [16]*.* State-specific data for this age group is unavailable. In spite of this low HPV vaccine coverage, recommendations from physicians remain suboptimal for all age groups [17].

While the choice whether or not to vaccinate adolescent usually falls to their parents, young women who are eligible to receive the vaccine are able to make the decision for themselves. These women are responsible for their *own* health, so their attitudes towards receiving the HPV vaccine and their decision-making processes may be different from those of the parents of young adolescents. There have been many studies on women’s beliefs and behaviors related to the HPV vaccine, but few studies have specifically focused on a state with low HPV vaccine initiation and completion rates [18–25].

The purpose of this study is to assess the demographic and attitudinal factors associated with HPV initiation and completion among 18–26 year old women in Utah. Our goal is to generate information to develop intervention programs to increase HPV vaccination in this age group in Utah.

## Methods

This study was approved by the University of Utah Institutional Review Board.

### Survey development

Previous studies have used Health Belief Model (HBM) constructs [26, 27] and/or Social Cognitive Model (SCM) factors [28, 29] to identify predictors of HPV vaccination intention. The HBM considers potential motivating factors such as perceived severity (an individual’s assessment of the seriousness of the condition), perceived susceptibility (an individual’s assessment of their risk of getting the condition), expected benefit (an individual’s assessment of the positive rewards of adopting the behavior), self-efficacy, and perceived barriers [30]. Whereas SCM also incorporates a person’s social environment, e.g., peer group, and observational learning on their health behaviors [29]. Each question included in our survey corresponded to a conceptual variable representing one facet in either HBM or SCM [29, 30]. Survey items were also motivated by use of directed acyclic graphs and a survey developed by other researchers and shared with permission [23, 31].

Our study questionnaire included six sections:

1. Attitudes about health,
2. Attitudes about vaccines,
3. Demographic information and history/family history of cancer,
4. Attitudes about reproductive health,
5. Attitudes about the HPV vaccine, and
6. Future (intended) HPV vaccine use.

### Data collection

**Fig. 1:**
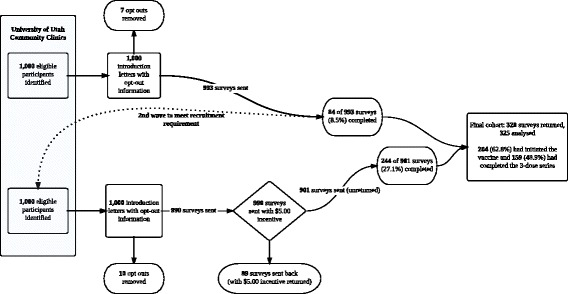
Survey sampling flow diagram (Utah, January-December 2013)

A Survey sampling flow diagram representing recruitment is presented in Fig. 1. We recruited participants from the University of Utah Community Clinics through the University of Utah Primary Care Research Network. An initial data query of potential participants was performed to identify young women age 18–26 years old who had a University of Utah Community Clinic visit in the 12 months preceding the query. Two groups of 1,000 were created. In the first group, we included 336 women who had at least one documented dose of the HPV vaccine and 664 unvaccinated women. In the second group we included 233 who had initiated the vaccine and 776 unvaccinated women. Potential participants were sorted by zip code and only those within the catchment areas for the University of Utah Community Clinics were included in the sample. Potential participants were mailed a letter briefly describing the project and given the opportunity to opt out. Remaining participants were mailed a letter describing the project in greater detail, a paper version of the survey, and a business reply envelope to return the survey.

Introduction letters with study opt-out information went out in two waves of 1,000. The initial wave of 1,000 opt-out letters was sent out in January 2013. After excluding nine opt-outs, we had a response rate of 84 of 991 surveys (8.5 %). The target response rate for analysis was between 300–325 completed surveys. In order to improve return rates, the implementation protocol was revised, following the Tailored Design Method proposed by Dillman, et. al. [32]. Modifications to the survey wave, according to the Dillman method, included:

- 1,000 Opt-out letters and cover letters were printed in high-resolution color and hand-signed,
- Surveys now included a 5$ bill thank you with return envelope, and
- Address verification services were used.

These revisions bolstered returns from 8.5 % to over 27.1 % (244 surveys completed of 901 delivered). All surveys were mailed between January and December 2013.

### Data analysis

Fisher’s Exact Tests and Logistic Regression were used to determine differences in demographics, initiation, and completion rates between the two waves of survey responses to assess appropriateness of pooling results. Summary statistics for participant characteristics were calculated by vaccination status. Principal component factor analysis with promax rotation was used for the attitude questions to derive useful attitude factor variables. Correlation between items in factor variables was assessed using Cronbach’s alpha (Table 3).

**Fig. 2:**
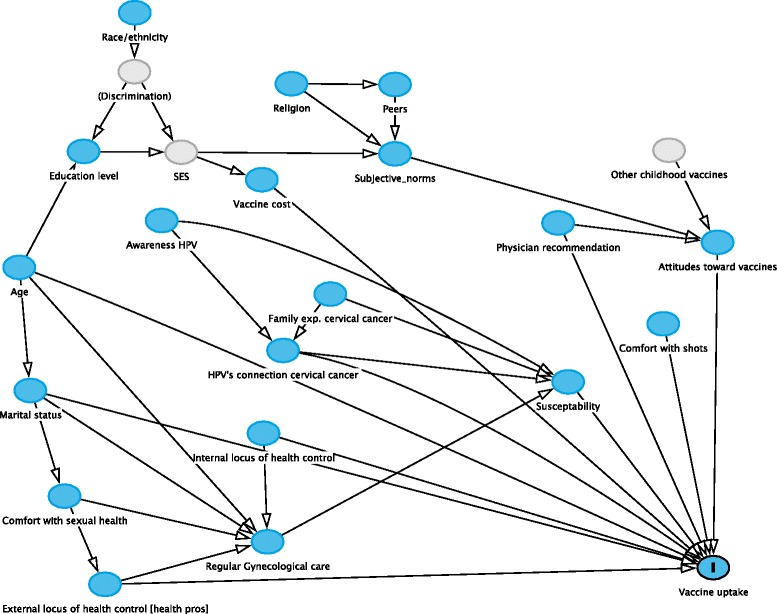
DAG showing potential cofounders of predictors of HPV vaccine uptake (Utah, January December 2013)

A Directed Acyclic Graph (DAG) (Fig. 2) was created to help identify and adjust for potential confounders and minimize bias assessing individual predictors of vaccine initiation (1+ dose) and series completion (3 doses) [33, 34]. An online tool, DAGitty (http://www.dagitty.net), was used to produce potential DAGs and derive minimally sufficient adjustment sets for predictor variables [35]. Univariate and multivariable logistic regression models were used to calculate odds ratios (ORs) to identify variables related to vaccine initiation and series completion.

Factor and Principal Component Analysis was performed using R (R Core Team, 2013). Descriptive statistics and univariate and multivariable logistic regression was performed using SAS software, Version 9.4. Results were considered statistically significant if *p <* 0.05.

## Results

A total of 84 of 993 surveys (8.5 %) from the first wave and 244 of 901 surveys (27.1 %) from the second wave were returned for a total of 328 of 1,983 returned (16.5 %). Three participants were excluded: two for having an age out of range and one for incompleteness of the questionnaire. The remaining 325 were included in the final analysis. There were no statistically significant differences between the two survey waves, and the only difference between wave one and wave two was wave two included a small incentive, but otherwise was drawn from the same population. Therefore, the two waves were pooled for the analysis.

Of these 325 participants, 204 (62.8 %) had initiated the vaccine series and 159 (48.9 %) had completed the 3-dose series. Of the 45 who had initiated, but not completed the vaccine series, 31 (70.5 %) said they intended to complete the series and 13 (29.6 %) said they did not (*one missing*). The mean age for those who initiated the vaccine was 22.4 years (s.d. 2.4 years), for those who completed the 3-dose series was 22.4 years (s.d. 2.4 years), and for those who did not receive any vaccine doses was 23.3 years (s.d. 2.3 years). Participant characteristics are presented in Table 1 and participants’ HPV vaccine related knowledge and attitudes are presented in Table 2.

**Table 1:** Characteristic of study participants (Utah, January-December 2013)

	No doses (*n =* 121) *n* (%)	1+ dose (*n =* 204) *n* (%)	3 dose completion (*n =* 159) *n* (%)
Age			
18–21.5 y.o.	28 (23.14)	82 (40.20)	65 (40.88)
22–24 y.o.	48 (39.67)	70 (34.31)	53 (33.33)
24.5–26 y.o.	45 (37.19)	52 (25.49)	41 (25.79)
Race/ethnicity			
Asian	9 (7.44)	8 (3.92)	8 (5.03)
Black or African American	2 (1.65)	4 (1.96)	3 (1.89)
Hispanic or Latina	9 (7.44)	15 (7.35)	10 (6.29)
White/Caucasian	96 (79.34)	167 (81.86)	133 (83.65)
Other	5 (4.13)	10 (4.90)	5 (3.14)
Highest level of education			
Up to or graduated high school	24 (19.83)	25 (12.25)	17 (10.69)
Some college, but no degree	39 (32.23)	90 (44.12)	72 (45.28)
College degree	46 (38.02)	77 (37.75)	60 (37.74)
Graduate school	12 (9.92)	12 (5.88)	10 (6.29)
Marital status			
Single, never married	66 (54.55)	165 (80.88)	132 (83.02)
(Ever) Married	54 (44.63)	38 (18.63)	26 (16.35)
Ever received a cancer diagnosis			
Yes	3 (2.48)	3 (1.47)	2 (1.26)
No	118 (97.52)	199 (97.55)	155 (97.48)
Know anyone who has had a cancer diagnosis			
Yes	95 (79.51)	178 (87.25)	140 (88.05)
No	26 (21.49)	26 (12.75)	19 (11.95)
Know anyone who has had cervical cancer			
Yes	9 (9.57)	24 (13.04)	18 (12.59)
No	85 (90.43)	160 (86.96)	125 (87.41)
Practice organized religion			
Yes	72 (59.50)	75 (36.95)	53 (33.54)
No	49 (40.50)	128 (63.05)	105 (66.46)
Religion guide your daily decisions			
Yes	57 (65.52)	51 (48.57)	38 (48.10)
No	30 (34.48)	54 (51.43)	41 (51.90)

**Table 2:** Participants’ attitudes about and knowledge relating to the HPV vaccine (Utah, January-December 2013)

	No doses (*n =* 119) *n* (%)	1+ dose (*n =* 202) *n* (%)	3 dose completion (*n =* 157) *n* (%)
Have you ever heard of human papillomavirus (HPV)?			
Yes	105 (86.78)	200 (98.04)	155 (97.48)
No	16 (13.22)	4 (1.96)	4 (2.52)
Do you know how HPV is spread?			
Yes	72 (59.50)	185 (90.69)	144 (90.57)
No	49 (40.50)	19 (9.31)	15 (9.43)
Have you ever heard of a relationship between HPV and cervical cancer?			
Yes	78 (65.00)	180 (88.24)	143 (89.94)
No	42 (35.00)	24 (11.76)	16 (10.06)
Have you ever heard of a vaccine to prevent HPV (e.g., Gardasil® or Cervarix®)?			
Yes	90 (75.00)	203 (100)	158 (100)
No	30 (25.00)	0 (0)	0 (0)
(If heard of vaccine) how important do you think the vaccine to help prevent cervical cancer is for you?			
Not at all important	10 (11.11)	5 (2.45)	2 (1.26)
Not very important	18 (20.00)	6 (2.94)	0 (0)
Somewhat important	27 (30.00)	50 (24.51)	33 (20.75)
Very important	35 (38.89)	143 (70.10)	124 (77.99)
Have you discussed the vaccine to help prevent cervical cancer with a doctor?			
Yes	34 (37.36)	201 (98.53)	158 (99.37)
No	57 (62.64)	3 (1.47)	1 (0.63)
Did a doctor recommend that you get the vaccine to help prevent cervical cancer?			
Yes	22 (55.00)	199 (98.03)	157 (99.37)
No	18 (45.00)	4 (1.97)	1 (0.63)

The factor analysis produced a total of seven attitude factors with good inter-item correlation, presented in Table 3. Table 4 shows the significant univariate predictors of vaccine *initiation* were lower age vs. older age: OR = 1.18 per lower year [95 % CI: 1.07–1.30]; marital status (being unmarried vs. married): OR = 3.62 [95 % CI: 2.18–5.99]; not practicing vs. practicing organized religion: OR = 2.4 [95 % CI: 1.49–4.0]; knowledge of HPV transmission: OR = 6.29 [95 % CI: 3.46–11.44]; known connection between HPV and cervical cancer: OR = 3.90 [95 % CI: 2.21–6.89]; known importance of vaccine (to help prevent cervical cancer): OR = 2.45 [95 % CI: 1.79–3.36]; strength of doctor recommendation: OR = 1.86 per Likert scale unit [95 % CI: 1.27–2.70]; and a binary indicator of whether a physician’s recommendation is influential: OR = 1.70 [95 % CI: 1.38–2.09]. These variables were all also significant predictors of *completion* of the 3-dose series (see Table 4). Minimal sufficient adjustment sets were derived from application of the DAG (see Fig. 2) to produce subsets of variables adequate to control for potential confounding. These subsets were used to produce adjusted estimates (see Table 4).

**Table 3:** Factor analysis results (Utah, January-December 2013)

Factor 1: Attitudes toward vaccines. [Cronbach’s alpha: 0.903 (*95 % CI: (0.880, 0.925))]*
• Vaccines are a good way to protect public health. • I do not like the idea of vaccines.^a^ • Vaccines are generally safe. • Vaccines are a way to take good care of myself now and in the future. • Vaccines are effective. • Vaccines are safe. In particular, HPV vaccine is safe.
Factor 2: Regular gynecological care. [Cronbach’s alpha: 0.856 *(95 % CI: (0.819, 0.892))*]
• Gynecological/pelvic exams are necessary to stay healthy. • I get a Pap test/Pap smear according to my doctor’s/health care provider’s advice. • It is very important to have an annual pelvic exam.
Factor 3: Comfort with sexual health (care). [Cronbach’s alpha: 0.754 *(95 % CI: (0.701, 0.807))*] • I am comfortable discussing sexual health issues with a doctor or nurse. • I am comfortable discussing sexual health issues with others such as family or friends. • I don’t mind getting a gynecological/pelvic exam.
Factor 4: External locus of health control 1: (medical professionals drive health). [Cronbach’s alpha:
0.720 *(95 % CI: (0.668, 0.772))*] • Having regular contact with my physician is the best way for me to avoid illness. • Whenever I don’t feel well, I should consult a medically trained professional. • Health professionals control my health. • Regarding my health, I can only do what my doctor tells me to do.
Factor 5: Internal locus of health control. [Cronbach’s alpha: 0.734 *(95 % CI: (0.680, 0.788))*]
• I am in control of my health. •The main thing which affects my health is what I myself do. •If I take care of myself, I can avoid illness. • If I take the right actions, I can stay healthy.
Factor 6: External locus of health control 2: (health matter of luck). [Cronbach’s alpha: 0.646 *(95 % CI: (0.570, 0.722))]*
• Luck plays a big part in determining how soon I will recover from an illness. • My good health is largely a matter of good fortune. • If it’s meant to be, I will stay healthy. Factor 7: Comfort with shots. [Cronbach’s alpha: 0.729 *(95 % CI: (0.660, 0.797))*] • I am not afraid of shots. • Shots are very painful.^a^

^a^ *Reverse coded (6 minus response)*

**Table 4:** Crude and adjusted odds ratios (95 % CIs) for predictors of vaccination initiation and completion and adjustment variables used in regression modeling (Utah, January-December 2013)

	Initiated		Completed		DAG-directed Adjustment Variables^a^
	Crude (95 % CI)	Adjusted (95 % CI)	Crude (95 % CI)	Adjusted (95 % CI)	
Physician rec. influential (Likert)	1.71 (1.39–2.11)	1.86 (1.46–2.36)	1.85 (1.46–2.34)	2.01 (1.53–2.63)	Factor 1: Attitudes toward vaccines, Age
Age (per year)	0.85 (0.77–0.94)	**	0.87 (0.79–0.95)	**	**
Education (ref: High school)					Age
Some college	2.22 (1.13–4.35)	2.33 (1.15–4.70)	2.38 (1.20–4.71)	2.53 (1.25–5.14)	
College graduate	1.61 (0.82–3.14)	2.72 (1.26–5.88)	1.79 (0.90–3.56)	3.07 (1.39–6.77)	
Graduate school	0.96 (0.36–2.55)	1.62 (0.55–4.76)	1.34 0.49–3.66)	2.26 (0.74–6.90)	
Ethnicity (white vs. non-white)	1.18 (0.67–2.07)	**	1.42 (0.81–2.49)	**	**
Marital status (ever vs never married)	0.28 (0.17–0.47)	0.31 (0.18–0.53)	0.30 (0.18–0.50)	0.32 (0.18–0.56)	Age, Factor 2: Regular Gynecological care, Factor 4: External locus of health control 1: (medical pros), Factor 5: Internal locus of health control
Practice Organized Religion (yes vs no)	0.40 (0.25–0.63)	0.41 (0.25–0.67)	0.38 (0.25–0.61)	0.40 (0.25–0.64)	Factor 1: Attitudes toward vaccines, Physician recommendation

For variables with **, they are unconfounded without adjustment and so adjustment variables are not needed

^a^Minimal sufficient adjustment set for estimating the total effect of variable on Vaccine uptake

**Fig. 3:**
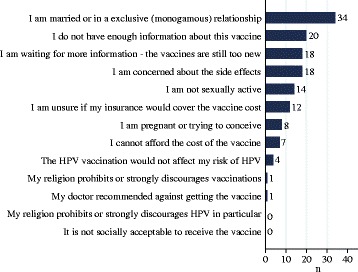
Reasons for not initiating or completing the HPV vaccine (Utah, January-December 2013)

The main reasons for not intending to initiate or complete the vaccine were waiting for more information/vaccine too new (*n =* 38), married or monogamous relationship (*n =* 36), cost of vaccine or unsure if insurance covers vaccine (*n =* 23), concern of side effects (*n =* 18), not sexually active (*n =* 14), and vaccine inconvenience (*n =* 7) (see Fig. 3).

## Discussion

The HPV vaccine has been available in the United States for eight years, yet only one third of adolescents have been fully immunized with all three recommended doses, and only one third of older eligible women (age 18–26 years old) have received just one dose of the 3-dose series [12, 15, 16]. HPV vaccine coverage varies substantially among states, with Utah having some of the lowest coverage rates in the U.S. [15]. Clearly, the Unites States is far from the Healthy People 2020 target of 80 % HPV vaccine coverage among eligible females, indicating an urgent need for interventions [15].

Resources are currently available to help increase HPV vaccination in Utah. In April 2007, Jon Huntsman, Sr. donated $1 million to the Utah Department of Health to educate Utahans about cervical cancer and provide low cost HPV vaccines to eligible women [36]. An additional $25,000 allocated by the Utah legislature was used for a public awareness media campaign and to inform physicians and other health care professionals about the HPV vaccine [36]. Additionally, the HPV vaccine is available for free or at low cost for girls age 9–18 years old through the Vaccines for Children Program and may be available for free or at low cost for women age 19–26 years old who have no insurance or insurance that does not pay for the vaccine thanks to Vaccine Patient Assistance Programs [37, 38]. The results of our study can help to focus these funds and resources to reach women who have not yet initiated and/or completed the HPV vaccination series.

Our study of young women age 18–26 years old found several correlates of vaccine initiation and completion that may be useful for future public health interventions for this population. Among these correlates were lower age, awareness of HPV transmission, knowledge of its connection to cervical cancer, belief in the importance of the HPV vaccine, and a physician recommendation (especially a strong recommendation) (Additional file 1: Figure S1). Marital status (being unmarried), practicing organized religion, and higher education were also significant predictors of vaccine initiation and completion. Cost, being in a monogamous relationship, and novelty of the vaccine were the main barriers against vaccination.

These findings echo previous studies that identified knowledge-attitude-practice gaps in the context of the HPV vaccine [39]. The differences we found between vaccinated women and unvaccinated women regarding risk beliefs (i.e., the vaccine is not personally relevant because they are in a monogamous relationship and the vaccine is too new and more information is needed) help explain why increasing uptake of the HPV vaccine requires targeted risk communication strategies [39]. Additionally, our study confirms one of the most ubiquitous finding in HPV vaccine research: the importance of a consistent and strong recommendation of the HPV vaccine from healthcare providers [23, 40, 41]. However, our findings run counter to an earlier study showing no association between marital status in their multivariable analysis [23]. This difference could be due to the particular interplay between marriage, age, and religiosity in the state of Utah.

There are limitations to the generalizability of the current study. The majority of participants were White/Caucasian (>80 %), had access to healthcare, and response rates were higher in the vaccinated vs. the unvaccinated, which may have introduced unmeasured response bias. Furthermore, cross-sectional study design prohibits assessment of causal relationships.

### Limitations

There are limitations to the generalizability of the current study. One of the primary limitations was the low survey return rate. Even after incentivizing a second wave of survey dissemination, response rates totaled well under 30 %. Therefore, our sample size was limited. Additionally, those who vaccinated were more likely to respond than those who did not vaccinate, leaving the potential for voluntary response bias. Furthermore, cross-sectional study design prohibits assessment of causal relationships.

The majority of participants were White/Caucasian (>80 %), had access to healthcare, and response rates were higher in the vaccinated vs. the unvaccinated, which may have introduced unmeasured response bias.

## Conclusions

The implications of these findings may help inform policies regarding HPV vaccination education among young women. For example, without this information programs might focus on awareness [36, 42–44], but the results of this study illustrate that the significance of awareness of HPV as a predictor of vaccine uptake has diminished over time and that programs should now focus on other variables (for example, strong and consistent physician recommendations). Additionally, our findings indicate the need for discussions of risk assessment tailored to the young adult population since young women are sure of their sexual behavior in a way parents may not be of their children’s. These interventions may use our results to take into account a patient’s education, religious affiliation and relationship status when having such conversations. Future research is needed to assess the impact these tailored interventions would have on bolstering HPV vaccination rates in the low vaccination state of Utah.

## Abbreviations

ACIP, advisory committee on immunization practices; CDC, centers for disease control; DAG, directed acyclic graph; HBM, health belief model; HPV, human papillomavirus; IRB, institutional review board; OR, odds ratio; SCM, social cognitive model

## Additional file

Additional file 1: Figure S1. Influence of rhysician reccomendation and age on probability of initiating HPV vaccine series. (PDF 555 kb)
