# Supplementary figures and images for: Factors related to HPV vaccine uptake and 3-dose completion among women in a low vaccination region of the USA: an observational study
Source: BMC Womens Health. 2016 Jul 22;16:41. doi: 10.1186/s12905-016-0323-5 (PMC4957275; doi:10.1186/s12905-016-0323-5)

# Influence of Physician Rec. & Age

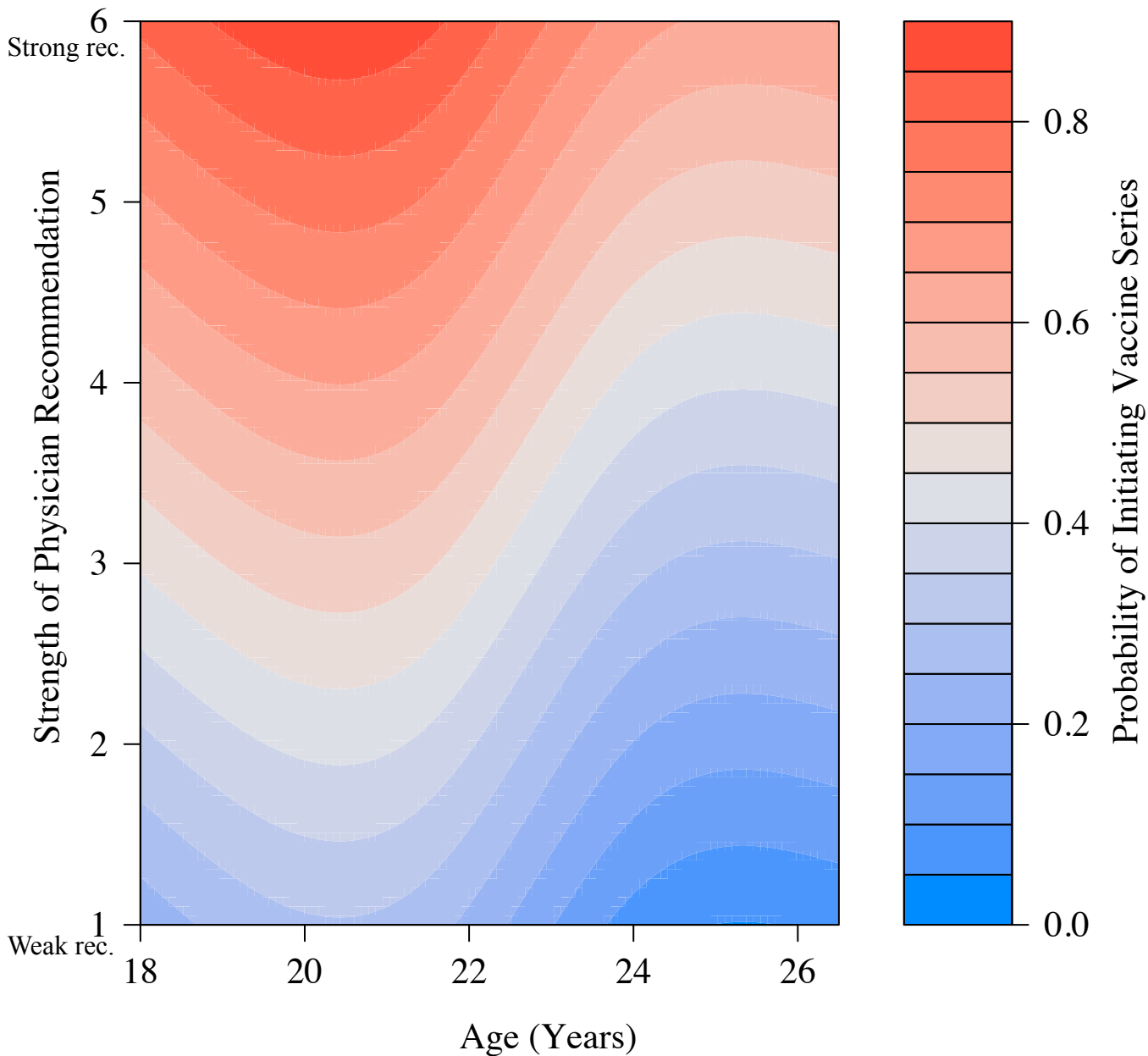

Supplement: Additional file 1: Figure S1. — Influence of rhysician reccomendation and age on probability of initiating HPV vaccine series. (PDF 555 kb) [file 12905_2016_323_MOESM1_ESM.pdf]
